# Sensory Processing, Perceived Stress and Burnout Symptoms in a Working Population during the COVID-19 Crisis

**DOI:** 10.3390/ijerph19042043

**Published:** 2022-02-11

**Authors:** Frank van den Boogert, Pascalle Spaan, Bram Sizoo, Yvonne H. A. Bouman, Witte J. G. Hoogendijk, Sabine J. Roza

**Affiliations:** 1 Department of Psychiatry, Erasmus University Medical Center, 3015 GD Rotterdam, The Netherlands; f.vandenboogert@erasmusmc.nl (F.v.d.B.); p.spaan@erasmusmc.nl (P.S.); w.hoogendijk@erasmusmc.nl (W.J.G.H.); 2 Department of Research, Transfore, 7416 SB Deventer, The Netherlands; y.bouman@transfore.nl; 3 Center for Developmental Disorders, Dimence Institute for Mental Health, 7416 SB Deventer, The Netherlands; bram@orcat.nl; 4 Netherlands Institute for Forensic Psychiatry and Psychology, 3511 EW Utrecht, The Netherlands

**Keywords:** sensory processing, sensory profile, sensory sensitivity, stress, burnout, COVID-19

## Abstract

Although previous research suggests an association between sensory processing and perceived stress in a broad spectrum of mental health conditions, it remains unclear whether this phenomenon occurs independently from psychopathology. The present study investigated the association between sensory processing patterns, perceived stress and occupational burnout as a stress-related condition in a working population. We focused on different aspects of sensory processing and used the momentum of a particularly stressful period: during the first months of the global COVID-19 crisis. A total of 116 workers at a mental healthcare institution in The Netherlands completed the Adolescent/Adult Sensory Profile (AASP), the Perceived Stress Scale (PSS-10) and the Burnout Assessment Tool (BAT). Our results demonstrated that higher scores on sensory sensitivity and low registration were associated with higher scores on perceived stress and core burnout symptoms. Sensory hypersensitivity was also associated with more secondary burnout symptoms. Associations were not driven by underlying sensory-related disorders (e.g., ASD or ADHD). In conclusion, sensory processing difficulties are relevant predictors of stress and occupational burnout, also in healthy employees. This phenomenon warrants further attention, as relatively simple adjustments in working environment may possess important preventive effects.

## 1. Introduction

Sensory processing is defined as receiving, modulating, integrating and organizing sensory stimuli, and the behavioral response to these stimuli [1]. Within the work environment, this includes the processing of auditory and visual information and other sensory stimuli, underlining the relevance of, for instance, acoustics and lighting. Persons with sensory processing difficulties might miss sound input such as ringing phones or incoming persons, may show humming or whistling in their working environment, or may overreact to colleagues or clients who get to close, etc. In general, humans are neurobiologically programmed to adapt their responses to environmental stimuli, however, variability among the general population is high and some people seem to have more sensitive brains or more difficulties in adapting their behavior to stimuli from their environment. Profound difficulties in sensory processing can be part of several mental health conditions, or may be recognized as a distinct sensory processing disorder (SPD) [2]. Whereas most individuals present a variation in their sensory processing patterns, difficulties in sensory processing might predispose health implications and the development of psychopathology [3]. Neurodevelopmental disorders such as autism spectrum disorder (ASD) and attention-deficit/hyperactivity disorder (ADHD) are often associated with sensory processing difficulties—even leading to incorporation of hyper- and hyporeactivity to sensory stimuli in the diagnostic criteria of ASD (DSM-5) [4]. Difficulties in sensory processing are, however, not restricted to individuals with neurodevelopmental disorders, and may be recognized as part of a more transdiagnostic phenomenon.

Sensory processing difficulties have been associated in the healthy population also, with various functional problems. On an emotional level, sensory processing is associated with anxiety level [5], negative affect [6] and distress [7]. Moreover, hypersensitivity is positively correlated with sleeping quality [8] and pain perception [9]. Sensory processing difficulties have been shown to negatively impact well-being, life satisfaction, and quality of life [10]. As such, sensory processing difficulties may interfere with performance and participation in different life contexts, including work. Furthermore, it may well be that sensory processing difficulties drive exaggerated stress responses to environmental stimuli, including work stress.

The possible association between sensory processing and perceived stress has been investigated primarily within the context of psychopathology and, particularly, in neurodevelopmental disorders. In children with ASD, sensory processing is associated with either heightened or lower stress hormone (cortisol) levels, depending on the type of aspect of sensory processing [11]. Hypersensitivity for sensory stimuli, for example, was associated with higher cortisol levels and higher physiological arousal during play activities [12]. In children with ADHD, sensory sensitivity was found to moderate the activity of the hypothalamic–pituitary–adrenal (HPA) axis and to differentiate between subtypes of ADHD [13]. There are also indications that sensory sensitivity is a relevant factor for perceived stress and anxiety in individuals with autistic traits, i.e., also in individuals without a clinical disorder. Whether sensory sensitivity leads to exaggerated stress responses, or exaggerated stress responses leads to sensory sensitivity, is largely unknown [14]. In veterans with acquired exaggerated stress responses due to PTSD, light and noise sensitivity are associated with more avoidance, intrusive experiences and hyperarousal [15]. Also in children, various aspects of sensory processing are found to be associated with posttraumatic stress [16]. Although results from previous research suggest an association between sensory sensitivity and perceived stress in psychopathology, it remains unclear whether sensory processing patterns other than sensory sensitivity, such as sensory seeking or sensory avoiding behaviors or those registered as sensory stimuli [17], are relevant in this context, and whether this phenomenon occurs independently from psychopathology.

A well-known stress-related condition is occupational burnout. In short, this condition can be defined as exhaustion due to prolonged exposure to work-related problems [18]. Although exhaustion is indeed at the symptomatologic core of occupational burnout, Schaufeli, et al. [19] broadened this definition by including other core and additional dimensions: “a work-related state of exhaustion that occurs among employees, which is characterized by extreme tiredness, reduced ability to regulate cognitive and emotional processes, and mental distancing. These four core dimensions of burnout are accompanied by depressed mood as well as by non-specific psychological and psychosomatic complaints”. Whether sensory processing difficulties may serve as precursors or markers for occupational burnout, is largely unknown. Some studies described an association of sensory processing sensitivity with burnout [20,21]. However, these studies measured the concept of ‘sensory processing sensitivity’, which represents a proposed personality trait characterized by deeper cognitive processing of stimuli and heightened emotional reactivity, and is conceptually different from (or even unrelated to) the more neurological approach of sense-based processing and related sensory processing disorders (SPD) [22]. In other words, ‘being a hypersensitive person’ is not the same as ‘having sensory processing difficulties’, and the other way around. As yet, it is unknown if and how these sensory processing difficulties are part of occupational burnout symptomatology or sequelae.

Sensory processing difficulties can be transient over time and can be measured in detail for each sensory modality separately by fundamentally determining the individual neurological threshold (the intensity of sensory stimulation needed to evoke neural response) and the resulting behavioral response to the incoming sensory information [23], without directly taking personality traits, emotional reactivity and depth of cognitive processing into account. In the present study, we focused on this neurological approach, and studied the associations of these detailed modal sensory patterns to the complex symptomatology of perceived (job) stress and occupational burnout. We studied these associations in a (predominantly healthy) working population, however, during the stressful first months of the global COVID-19 crisis, which increased the statistical power to find a meaningful effect on occupational burnout. We hypothesized that the COVID-19 crisis would serve as a general risk factor for burnout, not only in ‘front line’ health care professionals, but also in other workers, due to higher job demands and lower job positives. Many workers were facing work overload, restricted work environment, and challenges to come up with effective strategies to continue their jobs. At the same time, they were facing lower job positives like enriching social interaction with colleagues and building competence.

## 2. Materials and Methods

### 2.1. Study Design

The Oostwest Project is an observational study in a population of employees at the Dimence Group Mental Health Care Institutions in The Netherlands. The main aim was to study the effects of a changing work situation due to the COVID-19 pandemic, such as the increased working from home and use of telepsychiatry, or social distancing within the work environment. Data were collected between June and August 2020. The project was approved by the institutional review board of Dimence Group (CWO-062020PSFB). All participants provided written informed consent after procedures were fully explained, in accordance with the World Medical Association Declaration of Helsinki. The survey was distributed to each participant using the GemsTracker online survey system. A total of 251 employees received our study information sheet and were invited to participate. After sufficient reflection time, 116 employees (*M_Age_* = 44.7, *SD_Age_* = 12.2; *N_Male_*/*N_Female_* = 33/83) agreed to participate in the study. In total, five participants did not complete all four parts of the survey. However, all 116 participants did complete the first part of the survey, including the Work Situation questionnaire as described below.

### 2.2. Measurements

The Adolescent/Adult Sensory Profile (AASP) [17,24,25], a 60-item self-report questionnaire, was used to measure responsiveness to various sensory stimuli and to identify sensory processing difficulties that may hinder daily functioning. The AASP is the most frequently used instrument model for this purpose in adults and adolescents with ASD, however is also suited for use in other populations [26]. The questionnaire produces four continuous subscale scores ranging from 15 to 75, representing the four quadrants of the Model of Sensory Processing [17,23]: low registration (i.e., under-registration, e.g., missing stimuli such as sound input or slowed responses), sensory seeking, sensory sensitivity, and sensory avoiding. Each subscale consists of 15 items, rated on a 5-point Likert scale from never (1) to always (5). The values of the alpha coefficients for the quadrant scores range between 0.64 to 0.78 [27], which indicates satisfactory internal consistency. Within the present sample, the calculated Cronbach’s alpha was 0.84. We used all four raw quadrant scores for our analyses. Additionally, we calculated an AASP sum score, with a range of 60 to 300. For descriptive purposes, we calculated reference scores based on the data published in the instrument’s manual [24].

The 10-item version of the Perceived Stress Scale (PSS-10) [28] was used to measure the degree to which individuals appraise daily life situations in the last month as stressful and consists of items measuring self-efficacy and helplessness. This short version of the PSS has demonstrated high validity and reliability [29,30]. Each item is answered on a Likert scale from ‘never’ (0) to ‘very often’ (4). The total score ranged from 0 to 40, with a higher score indicating greater stress. The perceived helplessness subscale (ranging from 0 to 24) and the perceived self-efficacy subscale (ranging from 0 to 16) have been calculated as well. The calculated continuous raw scores were used in our statistical analyses, as described below. For descriptive purposes, the raw scores were compared to the reference data for males and females, as described in the PSS-10 manual [28]. Within the present sample, the calculated Cronbach’s alpha was 0.87.

The Burnout Assessment Tool (BAT) was used to estimate burnout symptomatology [31]. This 33-item self-report questionnaire consists of six different subscales: exhaustion, mental distance, cognitive impairment, emotional impairment, psychological complaints, and psychosomatic complaints. In line with instructions in the manual, the first four core symptoms were interpreted both separately and combined into a core symptoms score. Additionally, the latter two subscales were interpreted separately and combined as a secondary symptoms score. The core symptoms score, secondary symptoms score and the individual subscale scores were compared to the reference group of Flemish workers, as published in the instrument’s manual [31]. This resulted in ‘no risk’, ‘at risk’ and ‘very high risk’ scores. The instrument demonstrated adequate reliability, as well as convergent and discriminant validity with other burnout measures [19]. Within the present sample, the calculated Cronbach’s alpha was 0.94.

We measured various demographic and work-related variables. The applied educational levels were lower, middle and higher, in line with Dutch governmental guidelines issued by Statistics Netherlands. The lower educational level included primary and special primary education, prevocational education, the first three years of senior general secondary education and pre-university secondary education and the lower secondary vocational training. The middle educational level included the upper secondary education, vocational training and middle management and specialist education. The higher educational level included the associate, bachelor, master and doctoral degrees. These variables were used for sample characteristics and for limitation of confounding effects. We used a self-assembled 15-item Work Situation questionnaire to measure the perceived changes in work situation since the start of the COVID-19 crisis in The Netherlands, in March 2020. All questions were answered on a 5-point Likert scale, ranging from ‘strongly decreased’ to ‘strongly increased’. Data resulting from this questionnaire were solely used for descriptive purposes.

### 2.3. Statistical Analysis

For all statistical analyses, we used IBM SPSS version 25.0 (IBM Corp., New York, NY, USA). For descriptive purposes, means and standard deviations were calculated for continuous covariates, determinants and outcome variables and frequencies for dichotomous variables. In addition, where available, reference group distributions in percentages were calculated for sensory processing and stress- and burnout-related variables. We used multiple linear regression models to analyze the associations of sensory processing (AASP raw quadrant scores and AASP total score) with stress and burnout as outcome variables (PSS perceived stress, BAT core symptoms and BAT secondary symptoms). Secondary analyses were conducted for the more detailed subscale scores of the PSS and BAT. To overcome multicollinearity problems (high condition index values (> 30) and unstable b-coefficients) between the four raw quadrants of sensory processing, we applied forward selection as model building strategy. Age, sex, and educational level were standard covariates in the models. In the regression analyses with PSS total and subscale scores as outcome variable, one influential case was excluded due to random answering of this specific questionnaire. All other applicable statistical assumptions were met. Effect sizes of the predictor variables are expressed in Cohen’s *f*^2^, calculated using the *R*^2^ and Δ*R*^2^ of each model and interpreted according to Cohen’s guidelines [32].

## 3. Results

### 3.1. Descriptive Statistics

General sample characteristics are presented in Table 1. The majority of participants were female. Most participants were higher educated. A small number of participants reported potentially sensory-related conditions, such as a diagnosis of ASD, ADHD, neurological disease or the use of drugs or medications with possible sensory related effects in the last 30 days.

**Figure 1:**
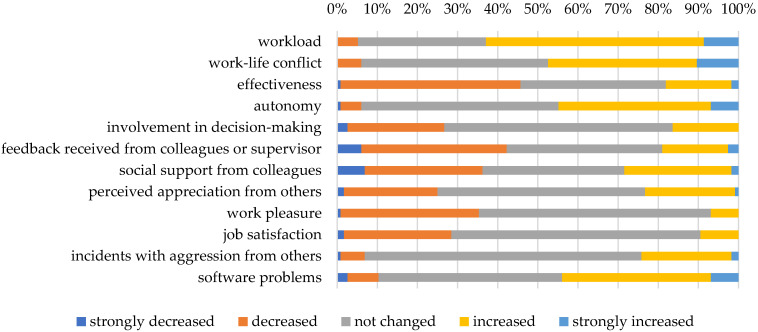
Perceived degree of changes in work situation since the Dutch COVID-19 measures of March 2020.

Perceived changes in work situation since the start of strict measures of social distancing, quarantine and lockdown in The Netherlands due to the COVID-19 pandemic in March 2020 are presented in Figure 1. Most of the participants in our sample experienced an increased level of workload. Moreover, a substantial part of the sample reported increased work-life conflicts and decreased effectiveness. Factors related to interaction with others partly showed decreased levels, although several employees (mainly working in inpatient settings) reported more involvement, feedback, social support, and appreciation by others. Approximately one third of our sample reported experiencing less pleasure in work, although job and financial security were perceived as fairly unchanged. Aggression-related incidents were reported as increased by a minority in the sample, mainly in the clinical setting. Finally, an increase in software problems was reported by almost half of the participants.

Means and standard deviations of all main measurements are presented in Table 2. In general, compared to the reference group of the AASP, our group of (predominantly healthy) workers was more often on the higher extremes of sensory sensitivity and sensory avoiding than expected. That means, more mental health professionals than expected reported to be hypersensitive to sensory stimuli and more mental health professionals than expected reported to avoid sensory stimulation. For perceived stress however, more participants in our sample than expected reported low stress (31% vs. 15%) and fewer participants than expected reported high stress (8% vs. 15%). On the burn-out scale, between 4.5% and 17.1% of participants in our sample reported the various (core or secondary) symptoms that indicate increased risk for burnout.

### 3.2. Regression Analysis

Results of our primary multiple regression analyses are presented in Table 3. In general, sensory processing problems were associated with higher levels of perceived stress, with more core burnout symptoms and with more secondary burnout symptoms, with medium to large effect sizes. Individuals with higher scores on low registration (having problems to notice or detect changes in sensory situations) and on sensory sensitivity (having problems of hypersensitivity), perceived more stress and reported more burnout symptoms.

Table 4 shows that sensory processing problems were associated with all stress- and burnout-related subscale scores separately. Largest effect sizes were seen for psychological and psychosomatic complaints and exhaustion in relation to sensory sensitivity and low registration.

As a post-hoc sensitivity analysis, the primary multiple regression analyses, as presented in Table 3, were repeated after exclusion of individuals with known medical conditions that are related to sensory problems (ASD, ADHD, neurological disease, or drug/medication use; *n* = 15), see Table 5. In the population without these medical conditions, we still found that low registration was related to perceived stress and core symptoms of burnout, and that hypersensitivity was related to secondary symptoms of burnout. Overall, associations remained statistically significant, although effect sizes were smaller.

## 4. Discussion

In the present study, we explored the association between sensory processing on the one hand and perceived stress and burnout symptomatology on the other hand in a working population during a highly demanding crisis period. Our results indicate that both sensory sensitivity and low registration are relevant in this context. Hypersensitivity for sensory stimuli was associated with more stress, more core symptoms of burnout, such as exhaustion, mental distancing and cognitive and emotional impairment, and more secondary symptoms of burnout, such as psychological and psychosomatic complaints. Under registration of sensory stimuli was associated with more stress and more core symptoms of burnout. Effect sizes of these associations were medium to large. Post-hoc sensitivity analysis revealed that correction for medical conditions, such as ASD, ADHD, or medication use, reduced effect sizes, however did not fully explain the found associations. On a more detailed level of stress perception, hypersensitivity was associated with lower levels of perceived self-efficacy, whereas under-registration of sensory stimuli were associated with higher levels of perceived helplessness.

Our results are in line with earlier studies on sensory sensitivity and perceived stress [11,12,13,14,15,16]. Whereas previous research was often done in clinical (child) populations of patients with neurodevelopmental symptomatology, with known medical conditions that influence sensory processing as well as coping with stress, our study shows that sensory processing is also related to perceived stress in healthy workers. Our results add to recent studies that focused on the association of sensory processing sensitivity and occupational burnout [20,21]. Risk of burnout is not only heightened by the more static vulnerability of personality traits such as ‘hypersensitive persons’, but is probably also increased in individuals with neurodevelopmental difficulties in sensory processing. Next, sensory processing difficulties could be considered as a potential part of burnout symptomatology or sequelae.

Although our study design limits causal inference, the more detailed measurement of sensory processing, stress and burnout enables us to cautiously explore hypothetical causal explanations. Largest effect sizes were seen in the associations of sensory sensitivity with psychological and psychosomatic complaints of occupational burnout. To some extent, these concepts might show overlap. It is tempting to speculate that psychosomatic complaints are related to interoception, the perception of bodily sensations, which is not assessed in the AASP questionnaire. Previous research suggests that the altered interoception may result from acute or chronic stress [33]. This (over)awareness of internal stimuli as a result of perceived stress might also apply to the other, more externally directed senses. This activation of all senses in reaction to stress seems evolutionary intuitive. On an item level, psychological complaints of burnout are operationalized by sleeping problems, anxiety, and problems with crowdedness and noise. Clearly, this last item might have conceptual overlap with sensory sensitivity to auditory stimuli. However, in addition to the suggested conceptual overlap, sensory processing difficulties could also be a transdiagnostic factor in relation to psychological complaints. In particular, experiencing heightened levels of sensory sensitivity resulting from chronic stress could eventually be exhausting and, thus, cause sleeping problems. Finally, under registration of external stimuli—low registration—could well be related to or perhaps result from problems with concentration and attention as signs of burnout-related cognitive impairment. More research is needed to unravel the exact underlying causal mechanisms in place.

The Oostwest Project data collection started in June and was completed in August 2020. These summer months cover the tail of the ‘first wave’ of coronavirus infections and government measures in The Netherlands, including social distancing, the closing of schools and childcare centers, cafes and restaurants, and sport clubs, and the urgent advice to work from home. As a result, some employees in the study sample were advised or obliged to work from home, while others could continue their clinical work in institutions with in-patient populations suffering severe mental illness, which could be exaggerated by the presence of the stressful psychosocial factor amid COVID-19. For many out-patient mental healthcare workers, it resulted in increased application of telepsychiatry, a worldwide phenomenon [34]. Our population reported increased problems on various aspects related to the individual work situation in this challenging, demanding, and downright tough period for caregivers and other employees. These circumstances could have resulted in more variance on stress- and burnout-related variables, whereas social distancing overall might decrease levels of sensory stimulation. Although we did not observe high numbers of employees facing occupational burnout during our measurement, it is possible that many continued to have high job demands and low job positives, eventually leading to more burnouts during the following phases of the pandemic.

Whether our results are generalizable to other (working and non-working) populations is largely unknown. Due to the healthy worker effect [35], morbidity in our source population was assumed to be decreased in comparison with the general population. However, reported sensory-related factors, such as ASD or ADHD, seem to be as prevalent as or even more prevalent in our sample in comparison to the general population. A second limitation is the potential risk for selection bias. Selective non-response could have led to both over- or underestimation of perceived stress and/or burnout symptomatology. It is however unknown whether the found associations with sensory processing difficulties might be different in those non-responded. Third, the risk for information bias cannot be ruled out, as we used self-report measures for both determinants and outcomes. Finally, we assume the inclusion of covariates and the executed sensitivity analyses have addressed potential confounding effects, however residual confounding can never be ruled out.

## 5. Conclusions

Our study underscores the relevance of sensory processing in general and specific sensory processing patterns in perceived stress, occupational burnout and potentially a broad spectrum of mental health conditions [3]. Problems in sensory processing are highly related to psychological and psychosomatic burnout complaints, which indicates that sensory processing, particularly hypersensitivity, is a transdiagnostic factor for psychopathology. More research is needed to thoroughly investigate the specific role of sensory processing in mental health. From a more clinical or occupational point of view, our results suggest a bidirectional approach. First, for employees who experience sensory processing difficulties, it is important to be aware of stress- and burnout-related problems. Second, employers do well to create a healthy working environment by paying attention to sensory processing patterns and difficulties in their employees and, as a result, regulate the levels of input on all senses. Employers can offer awareness on the subject, as well as personal (e.g., occupational diagnostics and therapeutic interventions) and environmental (e.g., acoustics, smell and lighting within the work space) prevention and support.

## Figures and Tables

**Table 1:** General sample characteristics (*n* = 116).

Descriptive		%
Age (M, SD)		44.7 (12.2)
Sex	Female	71.6
	Male	28.4
Educational level	Lower ^A^	3.4
	Middle ^B^	22.4
	Higher ^C^	74.1
Partner		86.2
Children		70.7
Profession	Psychologist/Psychiatrist	20.7
	Social Worker	18.1
	Nurse	12.9
	Other Clinical	6.0
	Security	6.9
	Consultancy and Management	24.1
	Secretarial and Administrative	11.2
Medical ^D^	ASD ^E^	2.6
	ADHD ^F^	4.3
	Drugs or medication in last 30 days ^G^	6.9
	Neurological diseases	4.3

^A^ Primary and special primary education, prevocational education, first three years of senior general secondary education and pre-university secondary education, lower secondary vocational training; ^B^ Upper secondary education, vocational training, middle management and specialist education; ^C^ Associate degree, bachelor degree, master degree, doctoral degree; ^D^ Variables known to be associated with sensory processing; ^E^ ASD = autism spectrum disorder; ^F^ ADHD = attention-deficit/hyperactivity disorder; ^G^ Drugs, which may influence sensory processing (e.g., antipsychotics, antidepressants, nausea medication or recreational drugs).

**Table 2:** Means and standard deviations of all involved total and subscale scores.

Instrument	Score	*M* (*SD*)	Comparison with Reference Group Data (%)				
AASP ^B^			−−	−	=	+	++
	Low registration	29.4 (6.0)	3.8	11.3	67.0	17.0	0.9
	Sensory seeking	50.1 (6.6)	2.7	10.0	70.0	17.3	0.0
	Sensory sensitivity	35.2 (8.2)	0.0	12.4	66.7	13.3	7.6
	Sensory avoiding	36.34 (8.5)	1.9	7.5	67.3	15.0	8.4
	Total score	150.9 (19.5)	^A^	^A^	^A^	^A^	^A^
PSS			<−1 SD		=		>+1 SD
	Perceived self-efficacy	11.6 (2.4)	^A^		^A^		^A^
	Perceived helplessness	6.1 (4.1)	^A^		^A^		^A^
	Perceived stress	10.5 (5.7)	30.6		61.3		8.1
BAT			Low risk		At risk		Very high risk
	Exhaustion	2.0 (0.6)	95.5		1.8		2.7
	Mental distancing	1.6 (0.5)	92.8		6.3		0.9
	Cognitive impairment	1.9 (0.5)	94.6		3.6		1.8
	Emotional impairment	1.7 (0.5)	82.9		13.5		3.6
	Core symptoms	1.8 (0.4)	94.6		4.5		0.9
	Psychological complaints	2.1 (0.6)	^A^		^A^		^A^
	Psychosomatic complaints	1.9 (0.7)	^A^		^A^		^A^
	Secondary symptoms	2.0 (0.6)	88.3		9.9		1.8

^A^ No reference group data available; ^B^ AASP reference values range from −− ‘much less than most people’ to ++ ‘much more than most people’.

**Table 3:** Multiple regression models with AASP raw quadrant and total scores, and stress- and burnout-related total scores.

	Perceived Stress	Core Burnout Symptoms	Secondary Burnout Symptoms
Low Registration	*b* = 0.262 [0.071, 0.453], *p* = 0.008	*b* = 0.025 [0.010, 0.040], *p* = 0.001	^A^
Sensory Seeking	^A^	^A^	^A^
Sensory Sensitivity	*b* = 0.156 [0.013, 0.300], *p* = 0.033	*b* = 0.012 [0.001, 0.023], *p* = 0.038	*b* = 0.041 [0.028, 0.053], *p* < 0.001
Sensory Avoiding	^A^	^A^	^A^
Effect Size ^B^	*f*^2^ = 0.26	*f*^2^ = 0.33	*f*^2^ = 0.41
AASP Total Score	*b* = 0.123 [0.071, 0.174], *p* < 0.001	*b* = 0.010 [0.006, 0.015], *p* < 0.001	*b* = 0.018 [0.013, 0.023], *p* < 0.001
Effect Size ^B^	*f*^2^ = 0.23	*f*^2^ = 0.26	*f*^2^ = 0.48

Note. All regression models were adjusted for covariates sex, age and educational level. ^A^ Variable excluded from analysis after application of the forward selection method. ^B^ Effect size of Δ*R*^2^ after adding predictor variable(s) in the second block in comparison to the covariates in the first block.

**Table 4:** Multiple regression models with AASP raw quadrant and total scores, and stress- and burnout-related subscale scores.

	Perceived Self-efficacy	Perceived Helplessness	Exhaustion	Mental Distancing	Cognitive Impairment	Emotional Impairment	Psychological Complaints	Psychosomatic Complaints
Low Registration	^A^	*b* = 0.260 [0.140, 0.380] *p* < 0.001	*b* = 0.025 [0.006, 0.043] *p* = 0.010	^A^	*b* = 0.039 [0.024, 0.054] *p* < 0.001	*b* = 0.029 [0.014, 0.045] *p* < 0.001	*b* = 0.023 [0.002, 0.045] *p* = 0.034	^A^
Sensory Seeking	^A^	^A^	^A^	^A^	^A^	^A^	^A^	^A^
Sensory Sensitivity	*b* = −0.095 [−0.143, −0.047] *p* < 0.001	^A^	*b* = 0.022 [0.008, 0.036] *p* = 0.002	*b* = 0.015 [0.002, 0.027] *p* = 0.020	^A^	^A^	*b* = 0.026 [0.010, 0.042] *p* = 0.002	*b* = 0.046 [0.032, 0.060] *p* < 0.001
Sensory Avoiding	^A^	^A^	^A^	^A^	^A^	^A^	^A^	^A^
Effect Size ^B^	*f*^2^ = 0.16	*f*^2^ = 0.19	*f*^2^ = 0.37	*f*^2^ = 0.06	*f*^2^ = 0.27	*f*^2^ = 0.14	*f*^2^ = 0.32	*f*^2^ = 0.42
AASP Total Score	*b* = −0.037 [−0.058, −0.017] *p* < 0.001	*b* = 0.085 [0.047, 0.123] *p* < 0.001	*b* = 0.014 [0.009, 0.019] *p* < 0.001	*b* = 0.006 [0.001, 0.012] *p* = 0.014	*b* = 0.012 [0.007, 0.016] *p* < 0.001	*b* = 0.007 [0.002, 0.012] *p* = 0.005	*b* = 0.017 [0.011, 0.023] *p* < 0.001	*b* = 0.019 [0.013, 0.025] *p* < 0.001
Effect Size ^B^	*f*^2^ = 0.13	*f*^2^ = 0.20	*f*^2^ = 0.28	*f*^2^ = 0.06	*f*^2^ = 0.23	*f*^2^ = 0.08	*f*^2^ = 0.36	*f*^2^ = 0.41

Note. All regression models were adjusted for covariates sex, age and educational level. ^A^ Variable excluded from analysis after application of the forward selection method. ^B^ Effect size of Δ*R*^2^ after adding predictor variable(s) in the second block in comparison to the covariates in the first block.

**Table 5:** Multiple regression models with AASP raw quadrant and total scores, and stress- and burnout-related total scores in healthy population.

	Perceived Stress	Core Symptoms Burnout	Secondary Symptoms Burnout
Low Registration	*b* = 0.337 [0.152, 0.522], *p* < 0.001	*b* = 0.030 [0.016, 0.045], *p* < 0.001	^A^
Sensory Seeking	^A^	^A^	^A^
Sensory Sensitivity	^A^	^A^	*b* = 0.036 [0.021, 0.051], *p* < 0.001
Sensory Avoiding	^A^	^A^	^A^
Effect Size ^B^	*f*^2^ = 0.16	*f*^2^ = 0.21	*f*^2^ = 0.26
AASP Total Score	*b* = 0.109 [0.051, 0.168], *p* < 0.001	*b* = 0.009 [0.005, 0.014], *p* < 0.001	*b* = 0.016 [0.010, 0.022], *p* < 0.001
Effect Size ^B^	*f*^2^ = 0.16	*f*^2^ = 0.20	*f*^2^ = 0.34

Note. All regression models were adjusted for covariates sex, age and educational level. ^A^ Variable excluded from analysis after application of the forward selection method. ^B^ Effect size of Δ*R*^2^ after adding predictor variable(s) in the second block in comparison to the covariates in the first block.

## Data Availability

The data presented in this study are available on request from the corresponding author. The data are not publicly available due to privacy regulations.
